# Supplementary material for: miRNAs-Set of Plasmatic Extracellular Vesicles as Novel Biomarkers for Hepatocellular Carcinoma Diagnosis Across Tumor Stage and Etiologies
Source: Int J Mol Sci. 2025 Mar 12;26(6):2563. doi: 10.3390/ijms26062563 (PMC11942138; doi:10.3390/ijms26062563)
Supplement: Supplementary file 1 [file ijms-26-02563-s001.zip › ijms-3503294 - Supplementary Data.pdf]

Supplementary Data

miRNAs-set of plasmatic extracellular vesicles as novel biomarkers for hepatocellular carcinoma diagnosis across tumor stage and etiologies

Francisco A. Molina-Pelayo#, David Zarate-Lopez#, Rosendo García-Carrillo, César Rodríguez-Beas, Ramón Íñiguez-Palomares, José L. Rodríguez-Mejía, Adriana Soto-Guzmán, Gabriela Velasco-Loyden, Mónica Sierra-Martínez, Adolfo Virgen-Ortiz, Enrique Sánchez-Pastor, Nancy E. Magaña-Vergara, Rafael Baltiérrez-Hoyos, Javier Alamilla, Victoria Chagoya de Sánchez, Adán Dagnino-Acosta, Enrique Chávez*, Luis Castro-Sánchez*

Table of Contents

Supplementary Material and Methods

Transmission Electron Microscopy 3

Dynamic Light Scattering (DLS) 3

Western Blot 3

Total RNA extraction 4

Isolation of miRNAs 4

Real-time qPCR for miRNAs 5

Microarrays for miRNAs Expression 5

Quantitative reverse transcription-polymerase chain reaction (RT-qPCR) for mRNA. 5

Supplementary Figures

Supplementary Fig. 1. miRNA expression in hepatic tissue of DEN-induced liver tumor rat model. 7

Supplementary Fig. 2. RNA quantification in EVs purified from HCC patients 8

Supplementary Fig. 3. Efficacy validation as diagnostic biomarkers for HCC patients 9

Supplementary Fig. 4. Efficacy validation as diagnostic biomarkers for HCC patients stratified by TNM staging 10

Supplementary Fig. 5. Efficacy validation as diagnostic biomarkers for HCC patients stratified by Edmondson-Steiner grading. 11

Supplementary Fig. 6. miRNA combinations analysis as a diagnostic panel for HCC detection. 12

Supplementary Tables

Supplementary Table 1. Clinicopathological features of the patients. 13
Supplementary Table 2. Average sizes and PDI of different HCC cells-secreted EVs. 14
Supplementary Table 3. Common miRNAs identified in HCC cell lines. 15
Supplementary Table 4. Common miRNAs identified in HCC cell lines-derived EVs. 16
Supplementary Table 5. DEN rat model features. 17
Supplementary Table 6. Oligonucleotide sequences employed for validation. 18
Supplementary Table 7. Average sizes and PDI of different EVs of plasma from DEN-induced liver tumor rat model. 19
Supplementary Table 8. CombiROC analysis of the five miRNAs proposed as biomarkers. 20
Supplementary Table 9. Markers in healthy group vs. HCC patients 21
Supplementary Table 10. Markers in healthy group vs Stage I 22
Supplementary Table 11. Markers in healthy group vs Stage II 23
Supplementary Table 12. Markers in healthy group vs Stage III 24
Supplementary Table 13. Markers in healthy group vs Stage IV 25

Transmission Electron Microscopy

Purified EVs were resuspended in 100 µl PBS with 1 µl 0.5 M EDTA pH 8.0. The EVs were then diluted 1:20 in PBS and 10.9 mM sodium citrate and placed on a formvar/carbon-coated copper TEM grid (300 mesh). The sample was stained with 2% phosphotungstic acid, dried overnight, and examined under a field-emission scanning electron microscope (JSM-7800 F, JEOL).

Dynamic Light Scattering (DLS)

Isolated EVs were resuspended in 1:100 dilution of 1X PBS pH 7.4 (10010023, Gibco, Thermo Fisher Scientific) and 0.5 M EDTA pH 8.0 (E6758-100G, Sigma-Aldrich), then diluted in 1:15000 of 10 mM HEPES pH 7.4 (H3375-100HG, Sigma-Aldrich). The size distribution was determined by DLS measurements using a Malvern Zetasizer NanoZS equipment (Malvern Instruments) set to 0.5 ηm resolution and sensitivity of 0.1 ppm to 40% w/v at 25ºC.

Western Blot

Proteins from liver tissue or purified EVs were extracted using 0.5% CHAPS total lysis buffer and subjected to Western Blot analysis. Briefly, whole-cell proteins were prepared with Laemmli sample buffer, separated by SDS-PAGE on 10% or 15 % polyacrylamide gels, and then transferred to PVDF membranes. The relative amount of protein was determined by immunodetection using monoclonal antibodies: anti-HSP90 α/β, anti-Alix (1:500, SC-13119, and SC-53540, 1:500; Santa Cruz Biotechnology, Inc. Dallas, TX, USA), anti-Flotilin-1, anti-Tsg-101 (1:500, BD-610821 and 1:1000, BD-612696, respectively, Franklin lakes, NJ, USA), anti-Calnexin (1:1000, SC-23954, Santa Cruz Biotechnology, Inc. Dallas, TX, USA), and GADPH (1:1000, ab9484, Abcam, Cambridge, UK). The blots were then incubated with horseradish peroxidase-conjugated anti-mouse secondary antibodies (1:5000; SC-516102; Santa Cruz Biotechnology, Inc. Dallas, TX, USA), and protein bands were visualized using a colorimetric substrate for peroxidase reaction with 1-Step Ultra TMB-Blotting Reagent (37574, Thermo Scientific, Waltham, MA, USA). Protein band densities were quantified and normalized to GADPH, and densitometric analyses were conducted using ImageJ software V1.54h 15 (NIH).

Total RNA extraction

25 to 30 mg of liver tissue from rats were processed with the Aurum Total RNA kit (Biorad No. 732-6820). First, the tissue was homogenized with 700 µL of lysis buffer (guanidinium thiocyanate and β-mercaptoethanol) and transferred to a 1.5 mL solvent-resistant conical microtube. The sample was centrifuged at 13,000 rpm for 5 min at room temperature. Subsequently, the supernatant was transferred to a new tube, and 700 µL of 70% ethanol was added and homogenized. Next, 700 µL of the mixture was transferred to an RNA affinity column and centrifuged at 13,000 rpm. After centrifugation, 700 µL of low stringency buffer was added to the column and then centrifuged at 13,000 rpm. Then, 80 µL of DNase was added to the column, and the sample was incubated for 15 min at room temperature. After incubation, a second wash was performed with 650 µL of high stringency buffer, centrifuged at 13,000 rpm. Finally, a last wash was performed with 700 µL of low stringency buffer and centrifuged at 13,000 rpm, followed by dry centrifugation at 13,000 rpm. To perform the elution, 20 µL of water free of RNAsas was used. Subsequently, the RNA concentration was quantified using the Quanti Fluor RNA System kit in a Quantus fluorometer (Promega). Finally, the RNA quality was analyzed in a QIAxpert high-speed UV/VIS microfluidic spectrophotometer (Qiagen).

Isolation of miRNAs

Cell cultures and purified EVs were resuspended in 700 µl Qiazol (79306, Qiagen, Germantown, MD) and incubated for 5 min at room temperature. Next, 140 µl of chloroform (C7559, Sigma, Burlington, MA, USA) was added and incubated for 5 min at room temperature. The samples were centrifuged at 12,000 × g for 15 min at 4 °C. The aqueous phase was collected and washed in a series of washing and centrifugation cycles at a constant 8,000 × g for 1 min at room temperature. First, 525 µl of absolute ethanol was added, and the mixture was centrifuged. Next, 700 µl RTW buffer was added, centrifuged, and decanted. Subsequently, 500 µl of RPE buffer was added, centrifuged, decanted, and repeated a second time but centrifuged for 2 min. Finally, 20 µl of RNase-free water was added and centrifuged at 8,000 × g for 1 min at 4 °C to elute the RNA. Subsequently, RNA concentration was quantified using the Quanti Fluor RNA System kit in a Quantus fluorometer (Promega,

Madison, WI, USA). Finally, RNA quality was analyzed in a QIAxpert high-speed UV/VIS microfluidic spectrophotometer (Qiagen).

Real-time qPCR for miRNAs

Cell culture and EV-contained miRNAs were reverse transcribed with the miScript II RT kit (Qiagen, Hilden, Germany) to obtain complementary DNA (cDNA) with the following thermal cycling conditions: 60 min at 37 °C followed by 5 min at 95 ºC. The cDNA was then amplified using miScript miRNA qPCR (Qiagen, Hilden) and the miRNAs oligonucleotides described in Table 1. The qPCR thermal cycling conditions were as follows: 15 min at 95 °C, followed by 40 cycles consisting of 15s at 94 ºC, 30 s at 55 ºC, and 30 s at 70 ºC. Data were extracted and analyzed using the Bio-Rad CFX Manager software (Bio-Rad). Gene reference stability analysis was performed to identify the most suitable internal control for all HCC models. Samples were analyzed in triplicate, and the data were analyzed and expressed as relative expression.

Microarrays for miRNAs Expression

The miRNAs from cell culture and EVs were subjected to reverse transcription to obtain cDNA, as described in the previous section. The cDNA was then amplified using a miScript miRNA Array Human Cancer PathwayFinder kit (331221 MIHS-102ZD, Qiagen, Hilden, Germany), which allows the evaluation of up to 84 cancer-related miRNAs. The qPCR microarray thermal cycling conditions were 15 min at 95 °C, followed by 40 cycles of 15 s at 94 ºC, 60 s at 55 ºC, and 30 s at 70 ºC. Data were extracted and analyzed using the Bio-Rad CFX Manager software (Bio-Rad). Heatmap and Venn diagrams were obtained using QCanvas 1.2 (CBiS, Seoul) and Venn Diagram tool (Bioinformatics & Evolutionary Genomics), respectively. Samples were evaluated by duplicate.

Quantitative reverse transcription-polymerase chain reaction (RT-qPCR) for mRNA.

The reverse transcription was performed with 0.5 μg of RNA in the iScript Synthesis kit (Biorad No. 170-8891) for 5 min at 25°C, 30 min at 42°C and 5 min at 85°C to obtain cDNA; then, it was mixed with the corresponding oligonucleotides (the designs were realized using primer3 and primer-BLAST from NCBI, and the qualities were evaluated by OligoAnalyzer Tool from IDT, OligoEvaluator from Sigma-Aldrich, uMelt, and Multiple Primer Analyzer from Thermo Fisher Scientific) with iQ SYBR Green (Bio-Rad) to perform real-time

quantitative PCR (RT-qPCR) on a CFX96 thermal cycler (Bio-Rad) with the following parameters: 3 min at 95°C and 40 amplification cycles (15 s at 95°C and 60 s at 60 ºC), followed by a melting curves analysis using a temperature from 55 to 95 ºC with an increment of 0. 5 ºC every 2-5 s/cycle. Data from qPCRs were obtained using the CFX manager program (Biorad) and processed using the comparative Ct method. Each sample was analyzed in triplicate and normalized with the levels of the reference gene (ΔCt) and compared against the experimental conditions considered as control (ΔΔCt) to subsequently obtain the relative expression ($2^{-\Delta\Delta Ct}$) as the mRNA expression level compared to the control condition.

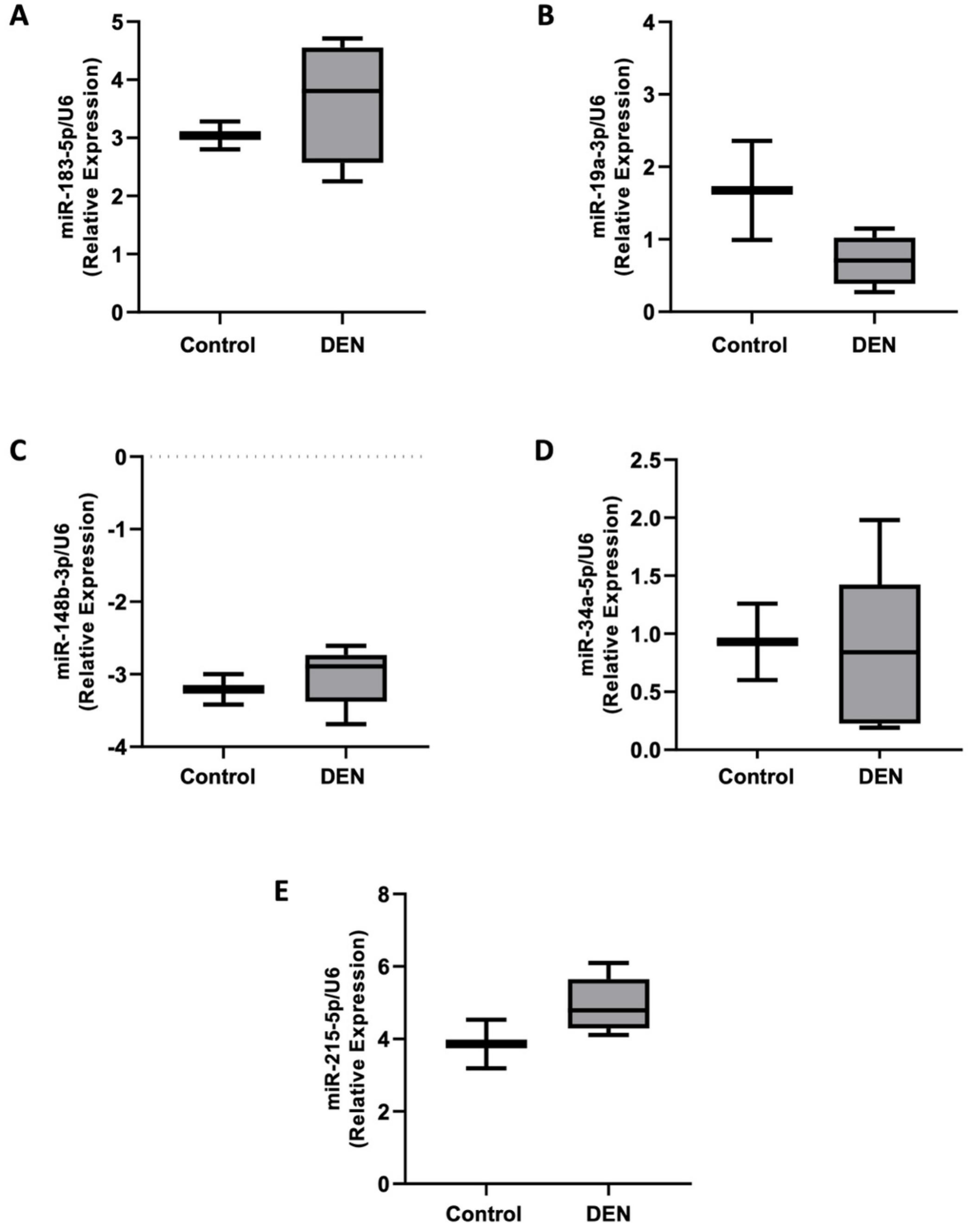

Supplementary Fig. 1. miRNA expression in hepatic tissue of DEN-induced liver tumor rat model. Total RNAs were obtained from the liver tissue of control and DEN-treated groups. The miRNA expressions in control and DEN-treated rats were determined by RT-qPCR **(A)**. Both groups calculated miRNA expressions as relative expressions and normalized U6 levels. miRNAs data expressions are represented in box plot graphs indicating the median and interquartile ranges.

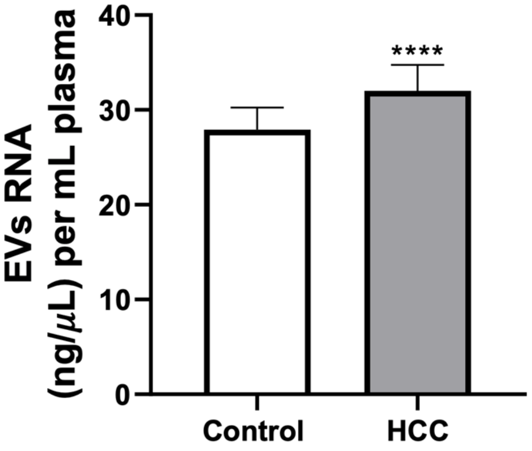

Supplementary Fig. 2. RNA quantification in EVs purified from HCC patients. Total RNAs were obtained from circulating EVs of plasma patients. The RNAs measurements in control subjects and HCC patients were determined by fluorometry (A). ****p<0.0001 vs. healthy subjects.

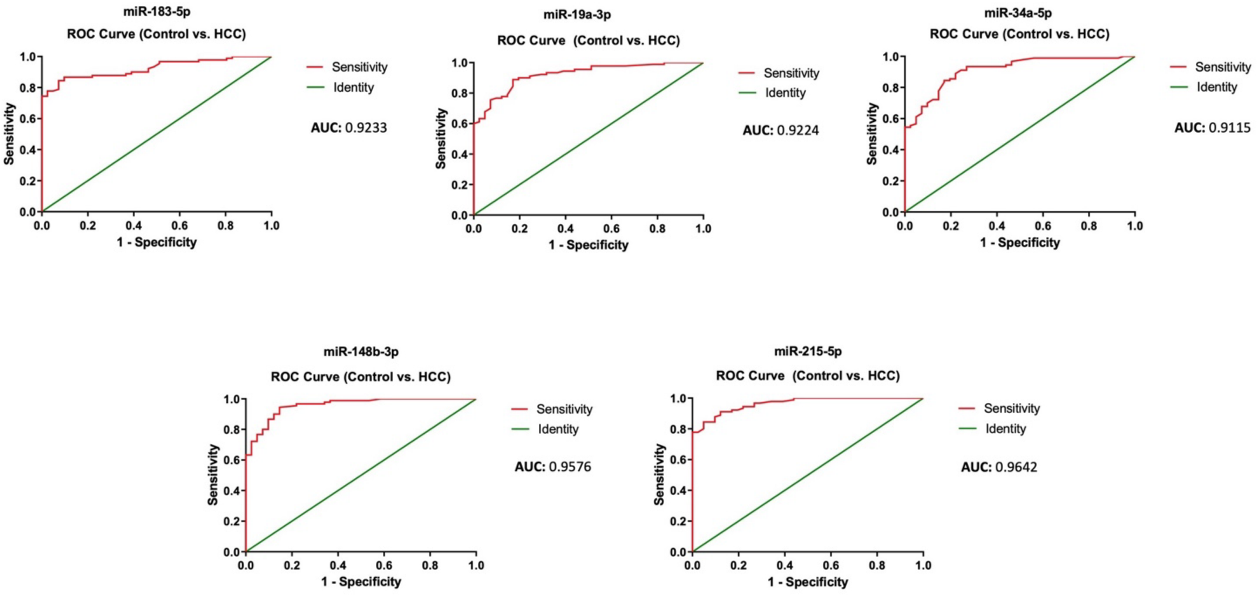

Supplementary Fig. 3. **Efficacy validation as diagnostic biomarkers for HCC patients.** ROC curves analysis from miRNA expression data of HCC patients compared to the control group.

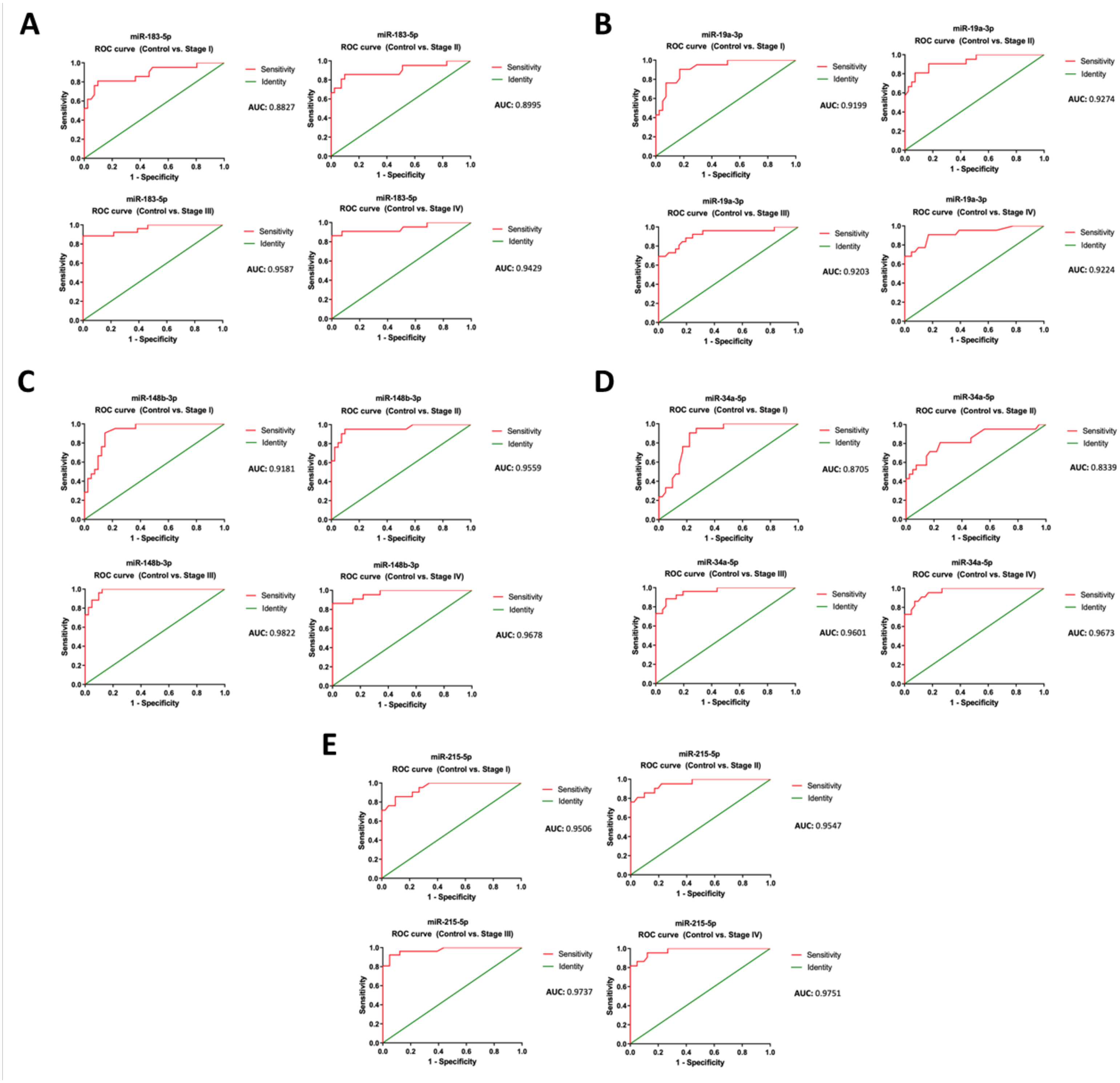

Supplementary Fig. 4. Efficacy validation as diagnostic biomarkers for HCC patients stratified by TNM staging. ROC curves for analyzed miRNA in HCC patients with different tumoral TNM stages compared to a control group.

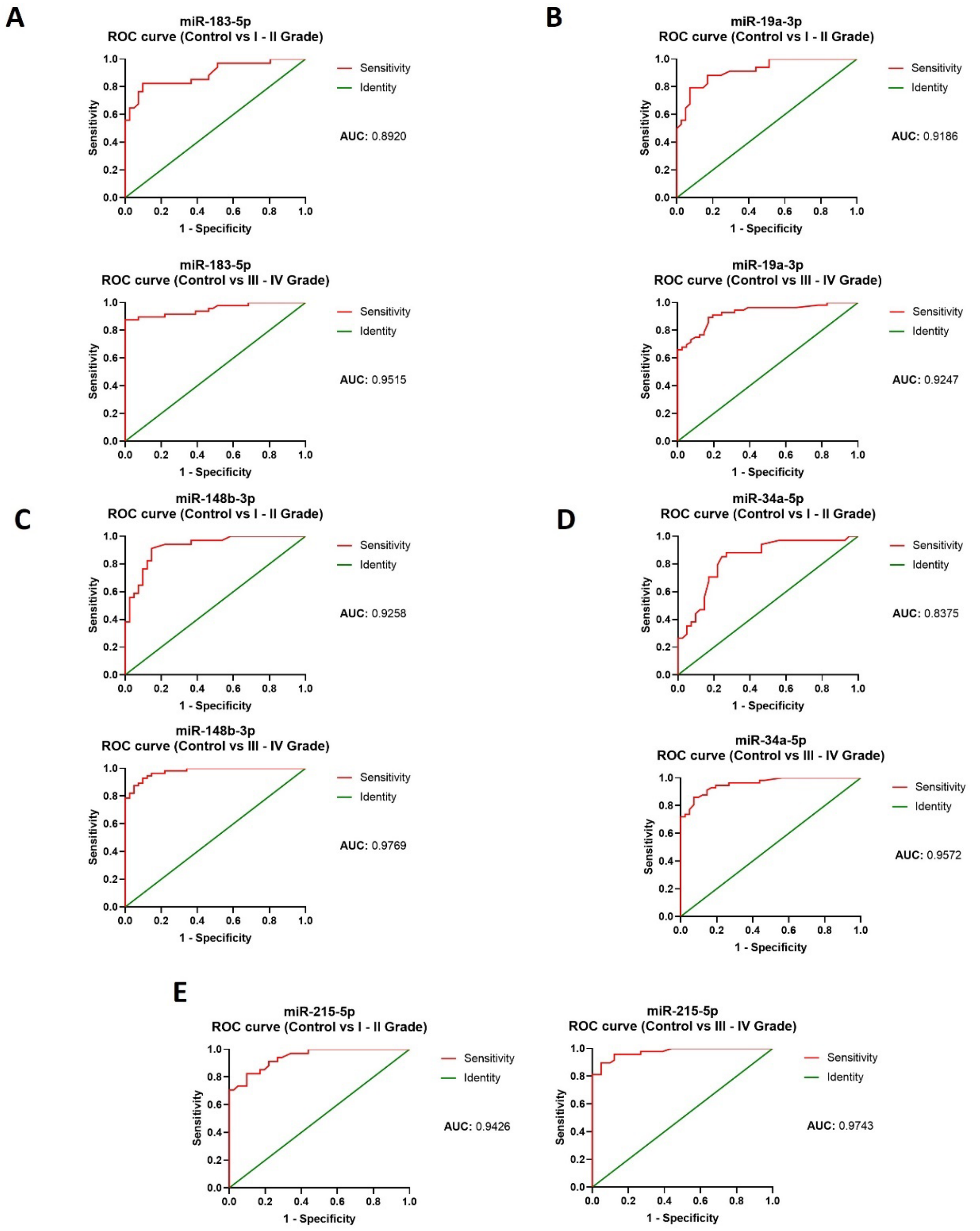

Supplementary Fig. 5. Efficacy validation as diagnostic biomarkers for HCC patients stratified by Edmondson-Steiner grading. ROC curves for analyzed miRNA in HCC patients with different tumoral grades compared to a control group.

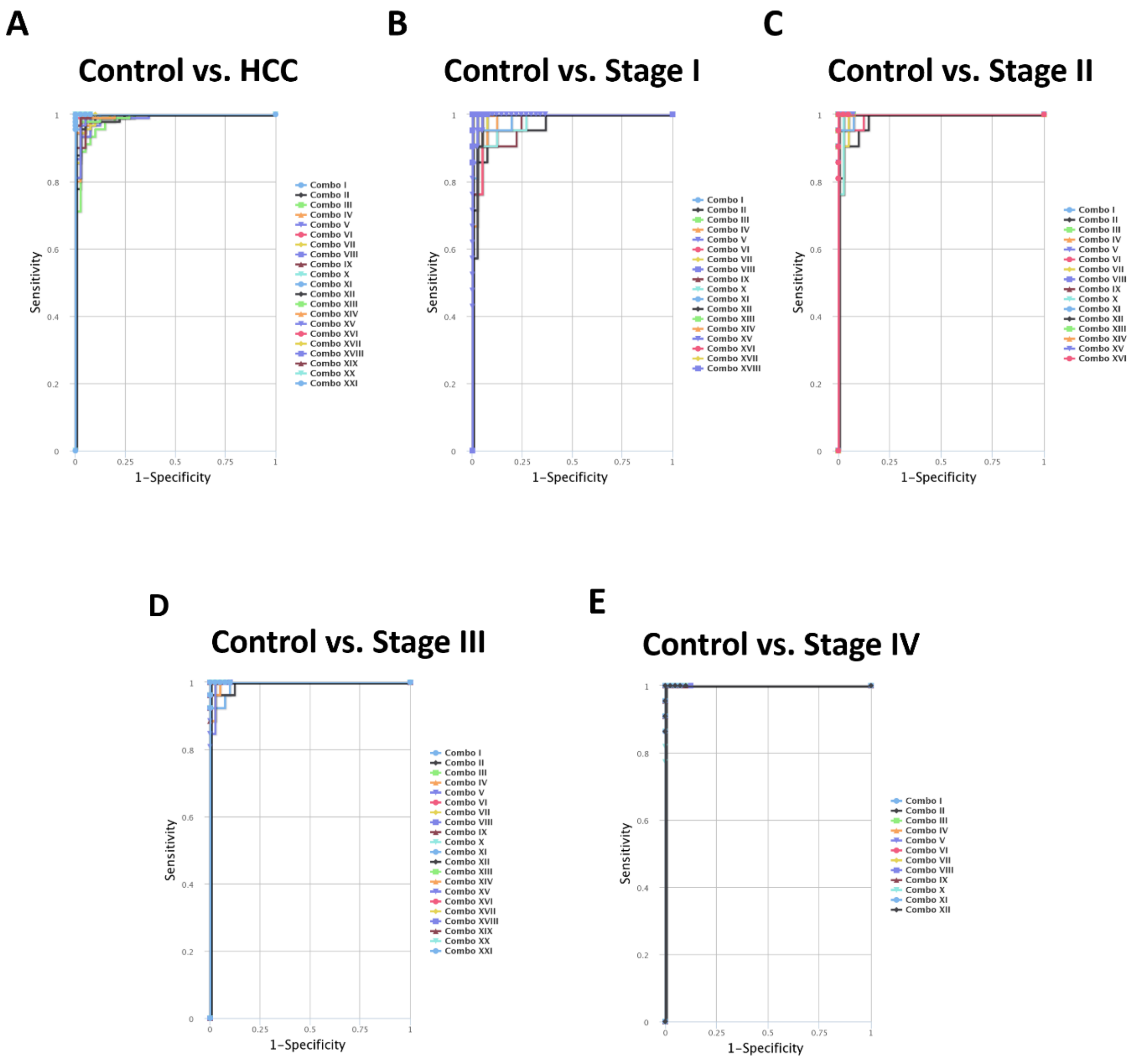

Supplementary Fig. 6. miRNA combinations analysis as a diagnostic panel for HCC detection. The miRNA data expression of all analyzed microRNAs was evaluated by the combiROC tool to obtain the optimal combinations for diagnostic accuracy. combiROC analysis: control vs. HCC (A), stage I (B), stage II (C), stage III (D), and stage IV (E).

Supplementary Table 1. Clinicopathological features of the patients.

Clinicopathological features	**Control** (*n*=41)	**Cirrhosis** (*n*=10)	**HCC** (*n*=90)
**Age (Years)**			
<50	20 (48.8%)	3 (30%)	10 (11.2%)
>50	21 (21.2%)	7 (70%)	80 (88.8%)
**Gender**			
Male	25 (60.1%)	8 (80%)	52 (57.8%)
Female	16 (39.9%)	2 (20%)	38 (42.2%)
**Etiology**			
HBV	0 (0%)	0 (0%)	18 (20%)
HCV	0 (0%)	4 (40%)	35 (38.8%)
Ethanol	0 (0%)	5 (50%)	25 (27.8%)
Unknown	0 (0%)	1 (10%)	12 (13.4%)
**TNM stage**			
I	0 (0%)	0 (0%)	21 (23.3%)
II	0 (0%)	0 (0%)	21 (23.3%)
III	0 (0%)	0 (0%)	26 (28.9%)
IV	0 (0%)	0 (0%)	22 (24.5%)
**Edmondson-Steiner grade**			
I-II	0 (0%)	0 (0%)	34 (37.7%)
III-IV	0 (0%)	0 (0%)	56 (62.2%)
**Tumor size (cm)**			
<3	0 (0%)	0 (0%)	18 (20%)
>3	0 (0%)	0 (0%)	72 (80%)
**AST (U/L)**			
<40	41 (100%)	0 (0%)	0 (0%)
>40	0 (0%)	10 (100%)	90 (100%)
**ALT (U/L)**			
<40	41 (100%)	0 (0%)	0 (0%)
>40	0 (0%)	10 (100%)	90 (100%)
**AFP (ng/mL)**			
≤20	0 (0%)	10 (100%)	0 (0%)
≤200	0 (0%)	0 (0%)	32 (35.5%)
>200	0 (0%)	0 (0%)	58 (64.5%)
**Cirrhosis**			
Positive	0 (0%)	10 (100%)	60 (66.6%)
Negative	0 (0%)	0 (0%)	30 (33.4%)

Supplementary Table 2. Average sizes and PDI of different HCC cells-secreted EVs.

Cell line	Mean size ± SD (ηm) Peak 1	PDI ± SD Peak 1	Mean size ± SD (ηm) Peak 2	PDI ± SD Peak 2
THLE-2	49.57 ± 10.63	0.48 ± 0.052	352.3 ± 77.22	0.394 ± 0.021
PLC/PRF/5	88.33 ± 8.301	0.702 ± 0.05	363.6 ± 104.8	0.475 ± 0.136
C3A(HepG2)	55.97 ± 12.23	0.672 ± 0.079	378 ± 2.33	0.575 ± 0.079
SNU-449	77.91 ± 21.79	0.518± 0.016	305.6 ± 104.2	0.518 ± 0.016
SNU-475	51.28 ± 10.73	0.535 ± 0.127	328.1 ± 77.83	0.518 ± 0.016
SNU-423	87 ± 1.223	0.709 ± 0.027	676 ± 41.53	0.724 ± 0.001

Supplementary Table 3. Common miRNAs identified in HCC cell lines.

Combination	Total	Overexpressed miRNAs
PLC/PRF/5<br>C3A (HepG2)<br>SNU-475<br>SNU-423	1	hsa-miR-148a-3p
PLC/PRF/5<br>SNU-449<br>SNU-475<br>SNU-423	1	hsa-miR-196a-5p
PLC/PRF/5<br>C3A (HepG2)<br>SNU-449	1	hsa-miR-122-5p
PLC/PRF/5<br>SNU-449<br>SNU-475	2	hsa-miR-128-3p; hsa-miR-9-5p
SNU-449<br>SNU-475<br>SNU-423	1	hsa-miR-181a-5p
PLC/PRF/5<br>C3A (HepG2)	1	hsa-miR-215-5p
PLC/PRF/5<br>SNU-475	5	hsa-miR-214-3p; hsa-miR-126-3p; hsa-miR-181c-5p; hsa-miR-143-3p; hsa-miR-148b-3p
SNU-449<br>SNU-475	2	hsa-miR-181b-5p; hsa-miR-301a-3p
SNU-475<br>SNU-423	3	hsa-miR-100-5p; hsa-miR-10b-5p; hsa-miR-10a-5p
PLC/PRF/5	10	hsa-miR-21-5p; hsa-miR-124-3p; hsa-miR-184; hsa-miR-92a-3p; hsa-miR-127-5p; hsa-miR-27a-3p; hsa-miR-19a-3p; hsa-miR-32-5p; hsa-miR-210-3p; hsa-miR-191-5p
C3A (HepG2)	4	hsa-miR-140-5p; hsa-miR-200c-3p; hsa-miR-34a-5p; hsa-miR-205-5p
SNU-449	1	hsa-miR-134-5p
SNU-475	10	hsa-miR-27b-3p; hsa-miR-149-5p; hsa-miR-130a-3p; hsa-miR-1-3p; hsa-miR-193b-3p; hsa-miR-125a-5p; hsa-miR-218-5p; hsa-miR-15a-5p; hsa-miR-23b-3p; hsa-miR-132-3p

Supplementary Table 4. Common miRNAs identified in HCC cell lines-derived EVs.

Combination	Total	Overexpressed miRNAs
PLC/PRF/5<br>SNU-449<br>SNU-475<br>SNU-423	6	hsa-miR-148a-3p; hsa-miR-20b-5p; hsa-miR-17-5p; hsa-miR-21-5p; hsa-miR-18a-5p; hsa-miR-92a-3p
C3A (HepG2)<br>SNU-449<br>SNU-475<br>SNU-423	1	hsa-miR-127-5p
PLC/PRF/5<br>SNU-449<br>SNU-475	1	hsa-miR-122-5p
SNU-449<br>SNU-475<br>SNU-423	39	hsa-miR-181b-5p; hsa-miR-140-5p; hsa-miR-372-3p; hsa-miR-149-5p; hsa-miR-34c-5p; hsa-miR-373-3p; hsa-miR-144-3p; hsa-miR-193-3p; hsa-miR-205-5p; hsa-miR-181a-5p; hsa-miR-378a-3p; hsa-miR-218-5p; hsa-miR-133b; hsa-miR-181d-5p; hsa-miR-125b-5p; hsa-miR-142-5p; hsa-miR-27b-3p; hsa-miR-124-3p; hsa-miR-155-5p; hsa-miR-128-3p; hsa-miR-200c-3p; hsa-miR-138-5p; hsa-miR-96-5p; hsa-miR-184; hsa-miR-9-5p; hsa-miR-150-5p; hsa-miR-1-3p; hsa-miR-203a-3p; hsa-miR-143-3p; hsa-miR-135b-5p; hsa-miR-100-5p; hsa-miR-10b-5p; hsa-miR-125a-5p; hsa-miR-16-5p; hsa-miR-193a-5p; hsa-miR-301a-3p; hsa-miR-206; hsa-miR-27a-3p; hsa-miR-210-3p
PLC/PRF/5<br>SNU-423	1	hsa-miR-222-3p
SNU-449<br>SNU-475	8	hsa-miR-335-5p; hsa-let-7d-5p; hsa-miR-23b-3p; hsa-miR-214-3p; hsa-miR-7-5p; hsa-miR-134-5p; hsa-miR-29b-3p; hsa-let-7c-5p
SNU-449<br>SNU-423	8	hsa-miR-25-3p; hsa-miR-30c-5p; hsa-miR-181c-5p; hsa-miR-29a-3p; hsa-miR-15a-5p; hsa-let-7g-5p; hsa-miR-32-5p; hsa-miR-196a-5p
SNU-475<br>SNU-423	3	hsa-miR-15b-5p; hsa-miR-20a-5p; hsa-miR-191-5p
SNU-449	7	hsa-let-7f-5p; hsa-miR-98-5p; hsa-miR-146b-5p; hsa-miR-132-3p; hsa-miR-126-3p; hsa-let-7e-5p; hsa-miR-10a-5p
SNU-423	1	hsa-let-7i-5p

Supplementary Table 5. DEN-induced liver tumor rat model features.

Condition	Body weight (g)	Liver weight (g)	Spleen weight (g)	Liver weight/Body weight (ratio)	Lipid peroxidation (nmol MDA/mg protein)	GGT (µmol/min/L)	ALT (µmol//min/L)	Survival
Control (*n* = 3)	454.70±13.67	18.11±0.98	1.10±0.10	0.04±0.00	0.36±0.08	4.65±0.04	24.44±4.28	100%
DEN (*n* = 10)	463.00±10.33	26.69±5.66	2.40±0.75	0.05±0.01	1.99±0.17*	5.69±0.42	38.59±3.31*	50%

The values are expressed as data ± SEM with a *p-value <0.05.

Supplementary Table 6. Oligonucleotide sequences employed for validation.

Gen	Primers (Sequence 5' -> 3')
hsa/rno-miR-183-5p	Forward: TATGGCACTGGTAGGAATTCACT
	Reverse: Universal Primer (Qiagen ID: 2183300)
hsa/rno-miR19a-3p	Forward: TGTGCAAATCTATGCAAAACTGA
	Reverse: Universal Primer (Qiagen ID: 2183300)
hsa/rno-miR-148b-3p	Forward: TCAGTGCATCACAGAACTTTGT
	Reverse: Universal Primer (Qiagen ID: 2183300)
hsa/rno-miR-34a-5p	Forward: TGGCAGTGTCTTAGCTGGTTGT
	Reverse: Universal Primer (Qiagen ID: 2183300)
hsa/rno-miR-215-5p	Forward: ATGACCTATGAATTGACAGAC
	Reverse: Universal Primer (Qiagen ID: 2183300)
RNU6 (Univer-U6)	Forward: GCTTCGGCAGCACATA
	Reverse: AAAATATGGAACGCTICACG
rno-*Afp*	Forward: AGCGCATCCATTTCCTTCCT
	Reverse: TTCATTGCAGCCAACGCATC
rno-*Gpc3*	Forward: ATCCAGCCGAAGAAGGGAAC
	Reverse: CAGCACAGGGTGTCGTTTTC

Supplementary Table 7. Average sizes and PDI of different EVs of plasma from DEN-induced liver tumor rat model.

Group	Mean size ± SD (ηm) Peak 1	PDI ± SD Peak 1	Mean size ± SD (ηm) Peak 2	PDI ± SD Peak 2	Mean size ± SD (ηm) Peak 3	PDI ± SD Peak 3
Control	696 ± 66.97	0.522 ± 0.102	-	-	-	-
DEN	19.9 ± 3.887	0.455 ± 0.096	51.9 ± 15.3	0.584 ± 0.096	273.6 ± 65.02	0.396 ± 0.096

Supplementary Table 8. CombiROC analysis of the five miRNAs proposed as biomarkers. The markers are as follows: Marker 1: hsa-miR-19a-3p; Marker 2: has-miR-34a-5p; Marker 3: hsa-miR-148b-3p; Marker 4: has-miR-183-5p and Marker 5: hsa-miR-215-5p.

Group	Symbol	Markers	AUC	Sensitivity (%)	Specificity (%)	Accuracy (%)
Control vs. HCC	Combo XXI	Marker1-Marker2-Marker3-Marker4-Marker5	1	100	100	100
Control vs. Stage I	Combo XVIII	Marker1-Marker2-Marker3-Marker4-Marker5	1	100	100	100
Control vs. Stage II	Combo XVI	Marker1-Marker2-Marker3-Marker4-Marker5	1	100	100	100
Control vs. Stage III	Combo XXI	Marker1-Marker2-Marker3-Marker4-Marker5	1	100	100	100
Control vs. Stage IV	Combo XII	Marker1-Marker2-Marker3-Marker4-Marker5	1	100	100	100

Supplementary Table 9. Markers in healthy group vs. HCC patients: The markers are as follows: Marker 1: hsa-miR-19a-3p; Marker 2: has-miR-34a-5p; Marker 3: hsa-miR-148b-3p; Marker 4: has-miR-183-5p and Marker 5: hsa-miR-215-5p.

Symbol	Markers	AUC	Sensitivity (%)	Specificity (%)	Accuracy (%)
Combo I	Marker1-Marker5	0.993	98.90	92.70	95.80
Combo II	Marker2-Marker5	0.990	94.40	97.60	96.00
Combo III	Marker3-Marker4	0.982	88.90	97.60	93.25
Combo IV	Marker3-Marker5	0.991	94.40	97.60	96.00
Combo V	Marker4-Marker5	0.989	93.30	97.60	95.45
Combo VI	Marker1-Marker2-Marker3	0.999	100.00	97.60	98.80
Combo VII	Marker1-Marker2-Marker4	0.992	94.40	97.60	96.00
Combo VIII	Marker1-Marker2-Marker5	0.995	100.00	97.60	98.80
Combo IX	Marker1-Marker3-Marker4	0.995	98.90	95.10	97.00
Combo X	Marker1-Marker3-Marker5	0.998	97.80	97.60	97.70
Combo XI	Marker1-Marker4-Marker5	0.997	95.60	100.00	97.80
Combo XII	Marker2-Marker3-Marker4	0.992	95.60	97.60	96.60
Combo XIII	Marker2-Marker3-Marker5	0.995	96.70	100.00	98.35
Combo XIV	Marker2-Marker4-Marker5	0.997	98.90	97.60	98.25
Combo XV	Marker3-Marker4-Marker5	0.995	100.00	95.10	97.55
Combo XVI	Marker1-Marker2-Marker3-Marker4	0.999	98.90	100.00	99.45
Combo XVII	Marker1-Marker2-Marker3-Marker5	1	100.00	100.00	100.00
Combo XVIII	Marker1-Marker2-Marker4-Marker5	0.999	100.00	97.60	98.80
Combo XIX	Marker1-Marker3-Marker4-Marker5	0.999	96.70	100.00	98.35
Combo XX	Marker2-Marker3-Marker4-Marker5	1	100.00	100.00	100.00
Combo XXI	Marker1-Marker2-Marker3-Marker4-Marker5	1	100.00	100.00	100.00

Supplementary Table 10. Markers in healthy group vs Stage I: The markers are as follows: Marker 1: hsa-miR-19a-3p; Marker 2: has-miR-34a-5p; Marker 3: hsa-miR-148b-3p; Marker 4: has-miR-183-5p and Marker 5: hsa-miR-215-5p.

Control vs. Stage I					
Symbol	Markers	AUC	Sensitivity (%)	Specificity (%)	Accuracy (%)
Combo I	Marker1-Marker5	0.992	100.00	92.70	96.35
Combo II	Marker4-Marker5	0.970	85.70	97.60	91.65
Combo III	Marker1-Marker2-Marker3	0.998	100.00	97.60	98.80
Combo IV	Marker1-Marker2-Marker4	0.981	95.20	92.70	93.95
Combo V	Marker1-Marker2-Marker5	0.994	100.00	97.60	98.80
Combo VI	Marker1-Marker3-Marker4	0.988	100.00	95.10	97.55
Combo VII	Marker1-Marker3-Marker5	0.994	100.00	92.70	96.35
Combo VIII	Marker1-Marker4-Marker5	0.997	100.00	97.60	98.80
Combo IX	Marker2-Marker3-Marker4	0.974	90.50	97.60	94.05
Combo X	Marker2-Marker3-Marker5	0.981	90.50	100.00	95.25
Combo XI	Marker2-Marker4-Marker5	0.987	95.20	97.60	96.40
Combo XII	Marker3-Marker4-Marker5	0.987	100.00	95.10	97.55
Combo XIII	Marker1-Marker2-Marker3-Marker4	1	100.00	100.00	100.00
Combo XIV	Marker1-Marker2-Marker3-Marker5	1	100.00	100.00	100.00
Combo XV	Marker1-Marker2-Marker4-Marker5	0.997	100.00	95.10	97.55
Combo XVI	Marker1-Marker3-Marker4-Marker5	1	100.00	100.00	100.00
Combo XVII	Marker2-Marker3-Marker4-Marker5	1	100.00	100.00	100.00
Combo XVIII	Marker1-Marker2-Marker3-Marker4-Marker5	1	100.00	100.00	100.00

Supplementary Table 11. Markers in healthy group vs Stage II: The markers are as follows: Marker 1: hsa-miR-19a-3p; Marker 2: has-miR-34a-5p; Marker 3: hsa-miR-148b-3p; Marker 4: has-miR-183-5p and Marker 5: hsa-miR-215-5p.

Control vs. Stage II					
Symbol	Markers	AUC	Sensitivity (%)	Specificity (%)	Accuracy (%)
Combo I	Marker1-Marker2-Marker3	0.998	95.20	100.00	97.60
Combo II	Marker1-Marker2-Marker4	0.986	90.50	97.60	94.05
Combo III	Marker1-Marker2-Marker5	0.994	100.00	97.60	98.80
Combo IV	Marker1-Marker3-Marker4	0.997	95.20	100.00	97.60
Combo V	Marker1-Marker3-Marker5	0.999	100.00	97.60	98.80
Combo VI	Marker1-Marker4-Marker5	0.994	95.20	100.00	97.60
Combo VII	Marker2-Marker3-Marker4	0.992	100.00	95.10	97.55
Combo VIII	Marker2-Marker3-Marker5	1	100.00	100.00	100.00
Combo IX	Marker2-Marker4-Marker5	1	100.00	100.00	100.00
Combo X	Marker3-Marker4-Marker5	0.994	100.00	97.60	98.80
Combo XI	Marker1-Marker2-Marker3-Marker4	0.997	95.20	100.00	97.60
Combo XII	Marker1-Marker2-Marker3-Marker5	1	100.00	100.00	100.00
Combo XIII	Marker1-Marker2-Marker4-Marker5	1	100.00	100.00	100.00
Combo XIV	Marker1-Marker3-Marker4-Marker5	1	100.00	100.00	100.00
Combo XV	Marker2-Marker3-Marker4-Marker5	1	100.00	100.00	100.00
Combo XVI	Marker1-Marker2-Marker3-Marker4-Marker5	1	100.00	100.00	100.00

Supplementary Table 12. Markers in healthy group vs Stage III: The markers are as follows: Marker 1: hsa-miR-19a-3p; Marker 2: has-miR-34a-5p; Marker 3: hsa-miR-148b-3p; Marker 4: has-miR-183-5p and Marker 5: hsa-miR-215-5p.

Control vs. Stage III					
Symbol	Markers	AUC	Sensitivity (%)	Specificity (%)	Accuracy (%)
Combo I	Marker2-Marker3	0.993	92.00	100.00	96.00
Combo II	Marker2-Marker4	0.995	96.00	100.00	98.00
Combo III	Marker2-Marker5	0.997	100.00	98.00	99.00
Combo IV	Marker3-Marker5	0.996	100.00	95.00	97.50
Combo V	Marker4-Marker5	1	100.00	100.00	100.00
Combo VI	Marker1-Marker2-Marker3	1	100.00	100.00	100.00
Combo VII	Marker1-Marker2-Marker4	1	100.00	100.00	100.00
Combo VIII	Marker1-Marker2-Marker5	0.996	100.00	98.00	99.00
Combo IX	Marker1-Marker3-Marker4	1	100.00	100.00	100.00
Combo X	Marker1-Marker3-Marker5	1	100.00	100.00	100.00
Combo XI	Marker1-Marker4-Marker5	1	100.00	100.00	100.00
Combo XII	Marker2-Marker3-Marker4	1	100.00	100.00	100.00
Combo XIII	Marker2-Marker3-Marker5	1	100.00	100.00	100.00
Combo XIV	Marker2-Marker4-Marker5	1	100.00	100.00	100.00
Combo XV	Marker3-Marker4-Marker5	1	100.00	100.00	100.00
Combo XVI	Marker1-Marker2-Marker3-Marker4	1	100.00	100.00	100.00
Combo XVII	Marker1-Marker2-Marker3-Marker5	1	100.00	100.00	100.00
Combo XVIII	Marker1-Marker2-Marker4-Marker5	1	100.00	100.00	100.00
Combo XIX	Marker1-Marker3-Marker4-Marker5	1	100.00	100.00	100.00
Combo XX	Marker2-Marker3-Marker4-Marker5	1	100.00	100.00	100.00
Combo XXI	Marker1-Marker2-Marker3-Marker4-Marker5	1	100.00	100.00	100.00

Supplementary Table 13. Markers in healthy group vs Stage IV: The markers are as follows: Marker 1: hsa-miR-19a-3p; Marker 2: has-miR-34a-5p; Marker 3: hsa-miR-148b-3p; Marker 4: has-miR-183-5p and Marker 5: hsa-miR-215-5p.

Control vs. Stage IV					
Symbol	Markers	AUC	Sensitivity (%)	Specificity (%)	Accuracy (%)
Combo I	Marker1-Marker3-Marker5	1	100.00	100.00	100.00
Combo II	Marker1-Marker4-Marker5	1	100.00	100.00	100.00
Combo III	Marker2-Marker3-Marker4	1	100.00	100.00	100.00
Combo IV	Marker2-Marker3-Marker5	1	100.00	100.00	100.00
Combo V	Marker2-Marker4-Marker5	1	100.00	100.00	100.00
Combo VI	Marker3-Marker4-Marker5	1	100.00	100.00	100.00
Combo VII	Marker1-Marker2-Marker3-Marker4	1	100.00	100.00	100.00
Combo VIII	Marker1-Marker2-Marker3-Marker5	1	100.00	100.00	100.00
Combo IX	Marker1-Marker2-Marker4-Marker5	1	100.00	100.00	100.00
Combo X	Marker1-Marker3-Marker4-Marker5	1	100.00	100.00	100.00
Combo XI	Marker2-Marker3-Marker4-Marker5	1	100.00	100.00	100.00
Combo XII	Marker1-Marker2-Marker3-Marker4-Marker5	1	100.00	100.00	100.00
